# Active case detection, treatment of falciparum malaria with combined chloroquine and sulphadoxine/pyrimethamine and vivax malaria with chloroquine and molecular markers of anti-malarial resistance in the Republic of Vanuatu

**DOI:** 10.1186/1475-2875-9-89

**Published:** 2010-04-06

**Authors:** Michael H Kinzer, Krisin Chand, Hasan Basri, Edith R Lederman, Augustina I Susanti, Iqbal Elyazar, George Taleo, William O Rogers, Michael J Bangs, Jason D Maguire

**Affiliations:** 1 U.S. Naval Medical Research Unit No.2, Kompleks P2P/PLP-LITBANGKES, Jl. Percetakan Negara No. 29, Jakarta Pusat 10560, Indonesia; 2 Vector Borne Diseases Control Programme, Ministry of Health, Private Mail Bag 042, Republic of Vanuatu; 3 Public Health & Malaria Control, Jl. Kertajasa, Kuala Kencana, Papua 99920, Indonesia

## Abstract

**Background:**

Chloroquine-resistant *Plasmodium falciparum *was first described in the Republic of Vanuatu in the early 1980s. In 1991, the Vanuatu Ministry of Health instituted new treatment guidelines for uncomplicated *P. falciparum *infection consisting of chloroquine/sulphadoxine-pyrimethamine combination therapy. Chloroquine remains the recommended treatment for *Plasmodium vivax*.

**Methods:**

In 2005, cross-sectional blood surveys at 45 sites on Malo Island were conducted and 4,060 adults and children screened for malaria. Of those screened, 203 volunteer study subjects without malaria at the time of screening were followed for 13 weeks to observe peak seasonal incidence of infection. Another 54 subjects with malaria were followed over a 28-day period to determine efficacy of anti-malarial therapy; chloroquine alone for *P. vivax *and chloroquine/sulphadoxine-pyrimethamine for *P. falciparum *infections.

**Results:**

The overall prevalence of parasitaemia by mass blood screening was 6%, equally divided between *P. falciparum *and *P. vivax*. Twenty percent and 23% of participants with patent *P. vivax *and *P. falciparum *parasitaemia, respectively, were febrile at the time of screening. In the incidence study cohort, after 2,303 person-weeks of follow-up, the incidence density of malaria was 1.3 cases per person-year with *P. vivax *predominating. Among individuals participating in the clinical trial, the 28-day chloroquine *P. vivax *cure rate was 100%. The 28-day chloroquine/sulphadoxine-pyrimethamine *P. falciparum *cure rate was 97%. The single treatment failure, confirmed by *merozoite surface protein-2 *genotyping, was classified as a day 28 late parasitological treatment failure. All *P. falciparum *isolates carried the Thr-76 *pfcrt *mutant allele and the double Asn-108 + Arg-59 *dhfr *mutant alleles. *Dhps *mutant alleles were not detected in the study sample.

**Conclusion:**

Peak seasonal malaria prevalence on Malo Island reached hypoendemic levels during the study observation period. The only *in vivo *malaria drug efficacy trial thus far published from the Republic of Vanuatu showed chloroquine/sulphadoxine-pyrimethamine combination therapy for *P. falciparum *and chloroquine alone for *P. vivax *to be highly efficacious. Although the chloroquine-resistant *pfcrt *allele was present in all *P. falciparum *isolates, mutant alleles in the *dhfr *and *dhps *genes do not yet occur to the extent required to confer sulphadoxine-pyrimethamine resistance in this population.

## Background

Malaria transmission is perennial throughout the Republic of Vanuatu, but seasonal in intensity. Its epidemiology has changed markedly since the first cases of chloroquine (CQ)-resistant *Plasmodium falciparum *were reported in the 1980s[1,2]. The efficacy of chloroquine against *P. vivax *has not been previously assessed in Vanuatu. Between 1988 and 2000, the national annual parasite incidence reported by passive case detection had decreased from 184 to 34 cases per 1,000 population after implementation of widespread insecticide-treated bed net distribution and new treatment guidelines[3]. In 1991, malaria treatment policy was changed from CQ monotherapy to CQ and sulphadoxine/pyrimethamine (SP) for uncomplicated *P. falciparum *infection. The regimen for *Plasmodium vivax *infection was changed from CQ + primaquine to CQ alone due to reports that glucose-6-phosphate dehydrogenase (G6PD) deficiency was relatively common in this population[4]. Elsewhere in the Asia-Pacific region, CQ+SP had already been found to be safe and well-tolerated but with varying efficacy [5,6]. In the Philippines, the combination was 87.5% effective compared to 30% for CQ alone[7], while in Central Java, Indonesia these drugs were 99% and 70% effective, respectively[8]. Mass treatment of the inhabitants of another isolated malaria endemic island in Vanuatu with CQ+SP plus primaquine successfully eliminated malaria without serious adverse events[9]. Some studies have shown little improvement in CQ+SP efficacy over SP alone in African and Asian regions with underlying SP and CQ resistance [10,11]. The efficacy of chloroquine against vivax malaria in this region has declined significantly over the last several decades with resistance rates as high as 22- 70% [12-15]. However, very little is known about anti-malarial drug resistance in Vanuatu. Genetic mutations associated with resistance to CQ and SP are well known. The mutant *pfcrt *76T allele, encoding a lysosomal transmembrane protein, facilitates the removal of CQ from the parasite lysosome, where it acts by inhibiting the detoxification and elimination of haemoglobin digestion products [16]. A combination of multiple point mutations in the *dhfr *gene confers resistance to pyrimethamine, a drug targeting its enzyme product dihydrofolate reductase [17]. Point mutations in the *dhps *gene are likewise associated with resistance to sulphadoxine through inhibition of parasite dihydropteroate synthase [18]. Point mutations in these latter two genes have been found in both resistant [19-21] and susceptible *P. falciparum *populations and several mutations are, therefore, likely to be required to confer resistance [22].

As emerging parasite resistance to older anti-malarials drives the revision of treatment guidelines and the adoption of newer, more expensive formulations with artemisinin derivatives, demonstration of the continued efficacy of CQ+SP through controlled clinical trials supports policies to conserve scarce healthcare resources [23]. At the time of this study, the seasonal epidemiology of malaria in central Vanuatu had not been characterized, nor had the efficacy of CQ+SP as first-line therapy been systematically evaluated, in spite of its widespread use over two decades. Similarly, the presence or absence of known genetic markers of plasmodial resistance to the current drug regimen in Vanuatu was unknown. Systematic policy-driven use of CQ+SP combination therapy may be changing the epidemiology and resistance patterns of *P. falciparum *among Vanuatan islanders. This study was designed to describe the current malaria situation on Malo Island in central Vanuatu where cross-sectional prevalence rates of 20% were observed in 2002 during the same season [4] by estimating the cross-sectional prevalence of malaria, the incidence of malaria during the purported period of high seasonal transmission and the efficacy of current malaria treatment standards by a 28 day follow-up *in vivo *treatment trial and analysing *P. falciparum *isolates for molecular markers of anti-malarial resistance.

## Methods

### Study site

**Figure 1:**
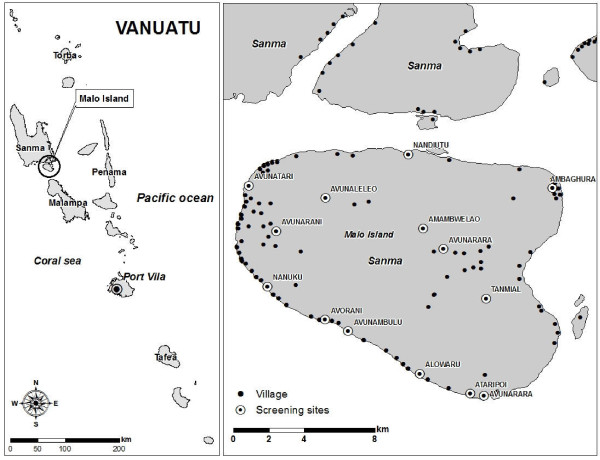
**Vanuatu map with location and distribution of participating Malo Island villages for mass blood survey, incidence study and malaria treatment trial, Feb - May 2005**.

The Republic of Vanuatu is an 80-island archipelago in the south-western Pacific with a population of approximately 200,000. Malaria is perennial with seasonal peaks and troughs, with a peak transmission season lasting from January to July, associated with hot and wet climate conditions, and declining transmission from north to south down the archipelago. The only known malaria vector is *Anopheles farauti *sensu stricto, which can breed in highly saline water [24,25]. Malo is centrally located (15°38'58"S, 167°05'57" E) within the Sanma Province just south of the larger island Espirito Santo (Figure 1). It has a stable population of approximately 4,600 inhabitants and has been characterized as having hypo- to meso-endemic malaria. A high season malariometric survey of 1,002 inhabitants in March of 2002 revealed a slide positive rate of 20% with a *P. falciparum *to *P. vivax *ratio of 3:1 and spleen rate (Hackett score 1-2) of 17.5% in children two to nine years of age[4]. In this same study, only 8% of *P. falciparum *infections and 4% of *P. vivax *infections were symptomatic on presentation, suggesting adequate transmission to induce semi-immunity.

### Study participants

Between February and May 2005, stations were established at 45 sites on Malo Island for mass blood screening of all inhabitants. Slides were stained and examined within 24 hours unless the participant was symptomatic, in which case the slide was read within two hours to expedite therapy. Eligible screened participants were enrolled into either an incidence study or a treatment trial. Infected individuals deemed ineligible for either were provided treatment free of charge according to Ministry of Health guidelines. Informed consent was obtained from adult participants and from the parents or legal guardians of minors. All work was performed in accordance with code 32 of Federal Regulations Part 219 (Protection of Human Subjects), U.S. Department of Defense, U.S. Navy (SECNAVINST 3900.39C) and Vanuatu Ministry of Health guidelines for the conduct of human use research. The protocols and informed consent processes were reviewed and approved by institutional review boards prior to initiating research.

### Incidence study

Based on previous surveys, approximately 85% of subjects were expected to become parasitaemic during a 16-week follow-up period. Using the sample size for estimating proportions formula, with an alpha of 0.05 (confidence) and an acceptable lower 95% confidence limit of 80%, 200 subjects were required to estimate cumulative incidence of asexual parasitaemia over the study period. Enrollment was offered to any person > 5 years of age with a negative blood smear on initial screening, selected into age and gender categories until closely matching recent Malo Island census data. In all participants, active surveillance consisted of a weekly Giemsa blood smear for 13 weeks or until diagnosis of first plasmodial infection. The first post-screening smear was performed on day 7 after the initial screening date and weekly thereafter. Any participant who presented with fever (> 37.5°F), chills, headache, myalgia, arthralgia, or nausea and/or vomiting between scheduled blood smears was microscopically evaluated for malaria. The primary endpoint of surveillance for each individual was their first documented parasitaemia, at which time they were treated per Ministry of Health guidelines. Each participant contributed person-weeks of follow-up until one of the following censoring events: 1) malaria parasitaemia, 2) voluntary withdrawal, 3) ingestion of anti-malarials, 4) loss to follow-up, 5) non-malarial illness prohibiting further participation, or 7) end of study.

### Treatment trial

Study procedures closely followed the World Health Organization (WHO) protocol for the *in vivo *28-day efficacy trial in low to moderate transmission areas for *P. falciparum *[26]. Similar methods were followed for *P. vivax *as previously performed at the Naval Medical Research Unit 2 [27]. Inclusion criteria were age ≥ 5 years, single species asexual parasitaemia with *P. falciparum *or *P. vivax*, parasite density > 400/μl (lower than WHO criterion of 1,000/μl), not pregnant, no consumption of anti-malarials during the prior week, no severe or complicated malaria (WHO definition), no other medical conditions requiring referral to a hospital for treatment, no ongoing antibiotic therapy or indication for antibiotic therapy, no history suggestive of hypersensitivity to CQ or SP and willingness to remain in the area until the completion of the study. Eligible volunteers provided a finger prick blood sample for repeat malaria smear, haemoglobin level and collection on Whatman No. 1 filter paper (Whatman International, Maidstone, Kent, UK) for polymerase chain reaction (PCR) testing. Women of childbearing age provided a urine sample for human chorionic gonadotropin testing (TestPack^® ^+Plus™ hCG Urine, Abbott, USA). All treatments were directly observed by study personnel. Uncomplicated *P. falciparum*- and *P. vivax *-infected participants received three daily doses of 10 mg/kg oral CQ (Resochin™ tablets, P.T. Bayer Indonesia) starting on the day of screening. *Plasmodium falciparum*-infected participants also received SP as one dose of 25 mg sulphadoxine + 1.25 mg pyrimethamine/kg body weight (Fansidar™, 25 mg pyrimethamine/500 mg sulphadoxine, Hoffman La Roche) at the same time as the first dose of CQ. If a participant vomited during the first thirty minutes after ingestion of medication, repeat dosing was provided. The second 10 mg/kg CQ dose was provided 24 hours later at the participant's home or place of work and the final 10 mg/kg dose 24 hours after the second dose. A health worker evaluated each participant at their home or work place and collected blood for malaria smears on days 0, 1, 2, 3, 7, 11, 14, 18, 21, and 28, and at any time a participant reported to the clinic with fever or other symptoms suggestive of malaria. Treatment failures were classified as early or late based on previously described WHO criteria [26].

### Laboratory methods

Thick and thin blood smears were stained with Giemsa and examined by a certified expert microscopist using 1,000× oil immersion light microscopy. Expert certification is determined by annual testing with requirement for 90% sensitivity and 100% specificity on a four species, 25 slide examination. At least 200 ocular fields were examined before a blood film was considered negative for *Plasmodium *sp. The microscopist recorded the number of asexual and sexual forms per 200 white blood cells in the thick smear. Parasitaemia was reported as parasites/μl with a conversion multiple of 40 (assumes a white blood cell count of 8,000/μl) for analysis. Genetic testing by PCR was limited to the treatment trial. Merozoite surface protein 2 (*msp-2*) genotyping was performed to aid in distinguishing *P. falciparum *recrudescence from re-infection in the treatment trial and *pfcrt*, *dhfr *and *dhps *PCR analysis was performed to identify point mutations associated with anti-malarial resistance. DNA was extracted from blood blot samples using previously described methods [28] followed by nested PCR and gene sequence-specific restriction-endonuclease digestion to detect *pfcrt *and *msp-2 *alleles as previously described [16,29]. *Dhfr *and *dhps *alleles were also characterized as previously described [30,31].

### Data analysis

All data were recorded on standardized written case report forms, double entered into a relational database in MS Access (Microsoft Inc., Redmond, WA) and analysed using SPSS software (SPSS Inc, Chicago, IL, USA). Analysis of mass blood screening data included standard descriptive statistics and stratified analysis by age and gender. The attributable fraction of fevers due to parasitaemia was also calculated [32]. Incidence rates were calculated in person-time as cases per person-week and cases per person-year based on the amount of time each participant contributed to follow-up. Time contributed by each individual was derived from the date of the last available blood smear result prior to withdrawal for any reason. The efficacy of each treatment arm was calculated as the percent of participants not requiring alternative therapy for recurrent parasitaemia during the 28-day follow-up. The first of two consecutive days without fever was considered the day of fever clearance. Treatment outcomes were also analysed by actuarial (life table) analysis as described elsewhere [33]. 95% confidence intervals for treatment efficacy rates and treatment failure rates were determined using the standard statistical formula 95% CI = estimator +/- (***z***_*(1-α/2) *_× standard error). All statistical tests were two-tailed with significance set at P < 0.05.

## Results and Discussion

### Mass blood screening

4,060 (88% of island population) adults and children were screened, of which 235 (6%) were infected with plasmodia based on Giemsa stained blood slides. Slide positivity rates by age group were 3% for children under 1 year, 10% for 1 to 5 years, 6% for 6 to 15 years, and 5% for over 15 years. Most cases were detected in the more heavily populated villages of Avunatari, Nanuku and Avunamblu (Figure 1). The spleen rate among 1,123 children (age 2-9 years) was 2.2% (95%CI 1.4 - 3.3) and consistent with hypo-endemic malaria. Fever rates among participants with *P. vivax *and *P. falciparum *were 20% and 23%, respectively. The attributable fraction of fevers due to parasitaemia in this population was only 5.2% (95% CI 4.2-6.2). The presence of fever in conjunction with a positive malaria smear correlated with parasite density in *P. falciparum *infections (F_1,34 _= 8.48, p = 0.006), but not in *P. vivax *infections (F_1,77 _= 1.89, p = 0.174). The *P. falciparum *to *P. vivax *ratio was 1.03 with only a single mixed infection identified and respective geometric mean parasite densities were 4,571 (95%CI 3111 - 6717) and 441 (95% CI 311 - 624). The percentage of those with *P. falciparum *or *P. vivax *gametocytaemia was 23% (95%CI 16 - 32) and 11% (95% CI 6 - 19), respectively. No *Plasmodium malariae *infections were found. Asexual stage parasites were found in 10 of the 28 (36%) infections with *P. falciparum *gametocytaemia and 100% of those with *P. vivax *gametocytaemia.

### Incidence study

**Figure 2:**
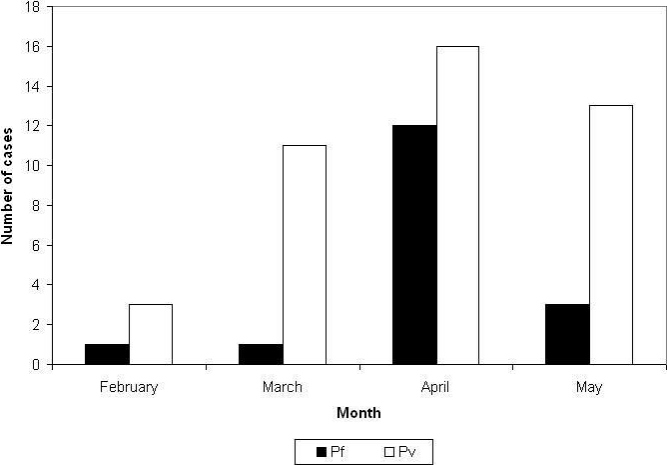
**Cases of malaria by species and month over 13 weeks of follow-up, Malo Island, Vanuatu, Feb-May 2005 (n = 203)**.

Two hundred and three individuals without malaria on initial screening were followed over 13 weeks for development of asexual parasitaemia, for a total of 2,303 person-weeks of follow-up. Sixteen week follow-up could not be achieved due to exhaustion of funds to sustain maintenance of personnel on site. Participants included 100 males and 103 females aged 5 to 74 years (mean 22) from the villages of Nandiuti (39%), Avunatari (36%), Nanuku (22%), Avunamblu (1.5%), Small Nanuku (1%) and Malo Pass (0.5%). The demographic make-up of the study group was similar to that of the general population (51% females, 55% children). Ninety-four percent of participants completed follow-up; of the 12 individuals who did not complete follow-up, seven were lost and five declined further participation. Seventeen (8%) participants developed *P. falciparum *and 43 (21%) developed *P. vivax *parasitaemia during follow-up. Children under the age of 15 were more likely to develop parasitaemia during follow-up than adults (OR = 3.3, 95%CI 1.6 - 7.1), an association that persisted whether those lost to follow-up were assumed to have been infected (OR = 2.1, 95%CI 1.1 - 3.9) or uninfected (OR = 3.7, 95%CI 1.8 - 7.7). The all-ages monthly incidence of *P. falciparum *peaked during April then rapidly declined, while *P. vivax *incidence rose in March, peaked in April and remained stable during May 2005 (Figure 2). The respective incidence densities for *P. vivax *and *P. falciparum *were 0.97 (95% CI 0.70 - 1.31) and 0.38 (95% CI 0.22 - 0.62) cases per person-year, with a combined incidence density of 1.36 (95% CI 1.04 - 1.75). A potential bias towards lower risk behaviour by enrolling only those who tested negative in a mass blood screening may lend to an underestimation of overall risk, however inclusion of participants with malaria who would have received treatment with a long half-life drug (CQ) on enrollment would have also contributed to an early underestimation of risk due to CQs prophylactic effect. The higher incidence observed for *P. vivax *compared to *P. falciparum *likely represents hypnozoite-related relapses from prior infection since radical cure with primaquine was not administered prior to enrollment in the incidence study. Only 15% of participants who developed malaria (47% of *P. falciparum *infections and 2% of *P. vivax *infections) had fever at the time of diagnosis, and there were no cases of severe malaria during the follow-up period. Geometric mean parasite densities were higher for *P. falciparum *than *P. vivax*, 1005/μl (range 362 - 2789) vs. 109/μl (range 75 - 185), but both densities were lower than observed in the mass blood survey, most likely due to early diagnosis through active screening in a population with high rates of asymptomatic infection.

### Treatment trial

**Figure 3:**
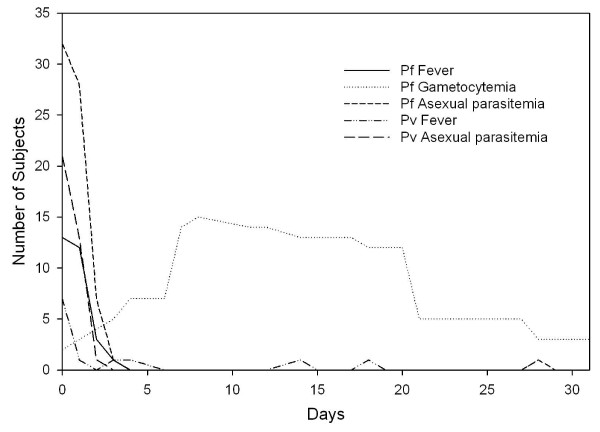
**Time course of fever, asexual parasitaemia, and gametocytaemia after treatment initiation in falciparum and vivax malaria patients, Malo Island, Vanuatu, Feb-May 2005**. *P. falciparum*, 33 cases; *P. vivax*, 21 cases

Since 1991, when Vanuatu implemented malaria treatment guidelines incorporating combination CQ+SP for *P. falciparum *malaria, no well-controlled study had been conducted to assess the regimen's effectiveness. Some studies have shown that combining CQ+SP may not enhance effectiveness of treatment over either medication alone [34-36]. In this study, 33 Malo Island participants with *P. falciparum *infections were treated with CQ+SP and 21 with *P. vivax *received CQ alone. Both drugs were given under direct observation and were well-tolerated without adverse events. The participants' age and gender distributions were representative of the general population. However, the target enrollment of 50 participants for each *Plasmodium *species was not reached due to a less-than-expected prevalence based on the 2002 survey, reducing the power of this study to detect treatment failures. Approximately one third of participants had documented fever and the majority of them reported at least one symptom associated with malaria (Table 1). During 28 days of follow-up, one participant was excluded on the first day because of the appearance of *P. vivax *parasitaemia after an initial diagnosis of *P. falciparum *mono-infection. None of those in the treatment trial were identified with an alternate species infection during the follow-up period. The average number of days of fever and asexual parasitaemia among *P. falciparum *infections were 1 and 2.1 days, respectively, and among *P. vivax *infection, 0.5 and 1.7 days (Figure 3). Asexual stage parasite clearance times were short (Figure 3). Only a single case of *P. vivax *had persistent parasitaemia past day one of therapy (day two parasite density 40/μl). Six cases of *P. falciparum *had detectable parasitaemia on day two but not beyond and only one persisted until day 3 at low density (120/μl). Gametocytaemia was common in *P. falciparum *infections throughout the follow-up period, as neither SP nor CQ are effective gametocidal agents[37] and SP is frequently associated with increasing gametocytaemia following therapy, an observation supported in this study on days six through 20 (Figure 3) [38]. Although CQ+SP was an effective treatment for asexual *P. falciparum *parasitaemia, this combination has the potential to increase transmission in the early post-treatment period. Gametocytaemia was not observed on initial diagnosis or at any time during the 28-day follow-up for any of the *P. vivax *cases.

**Table 1:** Demographic, parasitological and clinical parameters, malaria treatment trial, Malo Island, Vanuatu, Feb-May 2005 (n = 54)

	Treatment Group	
	
Variable	CQ for Pv	CQ+SP for Pf
Number of participants	21	33
Male : Female	7:14	18:55
Median age in years (range)	11 (5-53)	12 (5-51)
Mean weight in kg (range)	36.1 (15-80)	38.5 (16-66)
Spleen rate in children age 2-9 years (%)	1/15 (5%)	3/24 (9%)
Haemoglobin < 10 g/dl (%)	0 (0%)	1 (3%)
Temperature > 37.5°C (%)	7 (33%)	13 (39%)
Self-reported signs and symptoms* (%)	14 (67%)	31 (94%)
Geometric mean of parasite density/μl (range)	877 (499-1540)	5224 (3242-8418)

*Fever, rigors, headache, myalgia/arthralgia, nausea/vomiting, abdominal pain, and/or diarrhoea

The 28-day cure rate for *P. vivax *infections treated with CQ was 100% and that for *P. falciparum *treated with CQ+SP was 97%. Based on prior long standing criteria for conducting *in vivo *treatment trials by Naval Medical Research Unit 2 in Indonesia where chloroquine resistance rates for *P. falciparum *and *P. vivax *are high (8,34), a cut-off of > 400 asexual parasites per microliter of blood was set as the inclusionary parasite density for participation. This contrasts the more recent WHO standard of 1,000/μl (26), and may have led to overestimation of treatment efficacy. The single *P. falciparum *treatment failure occurred on day 28 and was classified as a late parasitological treatment failure (LPTF). On the day of recurrence, the participant was asymptomatic with a low parasite density of 200/μl and identical *msp-2 *genotypes on treatment days 0 and 28, supporting recrudescence vice re-infection. However, the allelic homogeneity of the infections sampled and low mean clone number of *msp-1 *in other samples from Vanuatu [39] suggest possible re-infection with the same strain. Although CQ mono-therapy proved highly efficacious against primary *P. vivax *infection in this study, without primaquine anti-relapse therapy, an unspecified portion of infections were expected to relapse later than the 28 days of follow-up observation period in this study. Additionally, the relatively small sample size precludes comparison of efficacy of CQ against *P. vivax *in Vanuatu with other regional locations with high rates of resistance. Larger studies are needed.

All 33 *P. falciparum *pre-treatment isolates from the treatment trial carried the Thr-76 *pfcrt *mutant allele as well as the double Asn-108 + Arg-59 *dhfr *mutant alleles and wild type Ala-16, Asn-51 and Ile-164 *dhfr *alleles. None of the isolates had *dhps *mutant alleles; all had wild type Ser- 436, Ala-437, Lys-540, Ala-581 and Ala-613. Although the chloroquine-resistant *pfcrt *allele was present in all samples tested, mutant allele combinations in the *dhfr *and *dhps *genes associated with clinical resistance were not seen. Point mutations associated with SP resistance were present, however, emergence of mutations in *Pfdhfr *usually precede those in *Pfdhps *[40]. Lack of *Pfdhps *mutations or triple *Pfdhfr *mutations in a population where SP has not been used extensively as monotherapy[41] may explain its continued efficacy in Vanuatu, and SP alone may currently be just as effective against *P. falciparum *as CQ+SP in Vanuatu. *Pvdhps *and *Pvdhfr *alleles for 18/21 pre-treatment isolates have been previously reported with high rates of triple (61% with S58R/T61M/S117T and 6% F57L/S117T/I173F)*Pvdhfr *mutations and a single quadruple (one with S58R/T61M/S117T/I173F) *Pvdhfr *mutation and only wild type *Pvdhps *alleles [22]. This suggests that indiscriminate use of CQ+SP over the preceding fourteen years has selected for *Pvdhfr *resistant alleles. Fortunately, chloroquine appears to remain efficacious against *P. vivax*, at least on this island.

## Conclusions

This study confirmed that malaria remains hypo- to meso-endemic on Malo and that *P. vivax *transmission is more than twice as likely as that of *P. falciparum *at the time of year of the 13-week observation. However, this ratio likely fluctuates throughout the year due to varying seasonal related transmission intensity of *P. falciparum *in Vanuatu [42]. The prevalence of malaria infection in 2005, 6%, was significantly less than that observed three years earlier (20%)[4]. The earlier survey also semi-quantitatively detected G6PD deficiency in 10% of individuals tested. High gametocyte carrier rates at the end of the peak transmission season and differences in fever and infection risk between children and adults suggest a persistent parasite reservoir throughout the presumed low transmission season. Gametocidal and liver stage-active drugs like primaquine may have a role for malaria control in this relatively isolated, low transmission area. Decisions about the use of primaquine for its gametocidal activity must be weighed against the natural background rate and clinical importance of G6PD deficiency in this population. A closer look at the impact of local public health measures (e.g., insecticide-treated bed net distribution and changes in treatment policy) and location-specific transmission dynamics is warranted to determine if they may play a more significant role in malaria transmission intensity than seasonal climatic patterns. A systematic entomological analysis to determine blood feeding frequency, times and location preferences (indoors or outdoors) for *An. farauti *s.s. and their relationship to bed net use would be logical next steps in improving understanding and control of malaria on Malo Island.

The combination of a cross-sectional mass blood screening survey, incidence study, and a treatment trial in a malaria-endemic island population provided the Vanuatu Ministry of Health with useful epidemiological and clinical data on malaria prevalence and transmission trends, as well as seasonal incidence and treatment efficacy fourteen years after a change in treatment guidelines in response to emerging CQ resistance. Additionally, the absence of *Pfdhfr *and *Pfdhps *allele combinations associated with SP resistance and maintenance of the CQ resistance conferring Thr-76 *pfcrt *mutant allele after extended use in combination with CQ was unexpected and potentially indicates a dominance of chloroquine's selective pressure over that of SP for *P. falciparum *in Vanuatu. The opposite appears to be true for *P. vivax *in Vanuatu, where the CQ + SP combination has likely been used to treat *P. vivax *in areas where limited diagnostic capabilities preclude confirmation of species, like Malo Island. Additional studies of these observations are warranted. A comprehensive approach to evaluating the state of malaria in specific locations like Malo Island as presented here can greatly assist health care policy decision makers by providing the information necessary to make data-driven treatment and prevention program adjustments and building a foundation for future malaria research and interventions.

## Competing interests

The authors declare that they have no competing interests.

## Authors' contributions

MHK contributed to the statistical analysis, and drafted the final manuscript, KC participated in the planning and implementation of the study, responsible for field data collection, and helped draft the manuscript, HB participated in the planning and implementation of the study, responsible for field data collection, and helped draft the manuscript, ERL participated in study design and coordination and served as field site supervisor during portions of study execution

AIS carried out the molecular genetic studies and helped draft the manuscript, IE created the databases, coordinated data management at the field site and performed the statistical analysis, GT conceived of the study and participated in its design and coordination, WOR contributed to statistical analysis and critically reviewed the manuscript, MJB conceived of the study, participated in its design and coordination, helped draft the manuscript and critically reviewed the manuscript, JDM conceived and designed the study, supervised study execution, analysed the data, helped draft the manuscript and critically reviewed the manuscript. All authors read and approved the final manuscript.

## Authors' information

Disclaimer: The assertions herein are the views of the authors and do not reflect official policy of the U.S. Department of the Navy, the U.S. Department of Defense or the U.S. government.

## References

[B1] Bowden DK Bastien P Douglas FP Muir JW Tambisari E Chloroquine-resistant *Plasmodium falciparum *malaria in Vanuatu Med J Aust 1982 2 561 562 6761561

[B2] Bastien P Saliou P Gentilini M [The chloroquine resistance of *Plasmodium falciparum *in Vanuatu (1980-1984): appearance, evolution, distribution](in French) Bull Soc Pathol Exot Filiales 1988 81 226 237 3046769

[B3] Vanuatu Ministry of Health Vanuatu Malaria Control Programme Data Book: 1983-1997 1998 Port Vila: Ministry of Health

[B4] Maguire JD Bangs MJ Brennan L Rieckmann K Taleo G Cross-sectional characterization of malaria in Sanma and Shefa Provinces, Republic of Vanuatu: malaria control implications P N G Med J 2006 49 22 31 18396609

[B5] McIntosh HM Greenwood BM Chloroquine or amodiaquine combined with sulfadoxine-pyrimethamine as a treatment for uncomplicated malaria--a systematic review Ann Trop Med Parasitol 1998 92 265 270 10.1080/00034989859825 9713541

[B6] Gogtay NJ Desai S Kadam VS Kamtekar KD Dalvi SS Kshirsagar NA A randomized, parallel-group study in Mumbai (Bombay), comparing chloroquine with chloroquine plus sulfadoxine-pyrimethamine in the treatment of adults with acute, uncomplicated, *Plasmodium falciparum *malaria Ann Trop Med Parasitol 2000 94 309 312 10.1080/00034980050034545 10945039

[B7] Bustos DG Canfield CJ Canete-Miguel E Hutchinson DB Atovaquone-proguanil compared with chloroquine and chloroquine-sulfadoxine-pyrimethamine for treatment of acute *Plasmodium falciparum *malaria in the Philippines J Infect Dis 1999 179 1587 1590 10.1086/314770 10228090

[B8] Maguire JD Lacy MD Sururi Sismadi P Krisin Wiady I Laksana B Bangs MJ Masbar S Susanti I Basuki W Barcus MJ Marwoto H Edstein MD Tjokrosonto S Baird JK Chloroquine or sulfadoxine-pyrimethamine for the treatment of uncomplicated, *Plasmodium falciparum *malaria during an epidemic in Central Java, Indonesia Ann Trop Med Parasitol 2002 96 655 668 10.1179/000349802125002310 12537627

[B9] Kaneko A Taleo G Kalkoa M Yamar S Kobayakawa T Bjorkman A Malaria eradication on islands Lancet 2000 356 1560 1564 10.1016/S0140-6736(00)03127-5 11075770

[B10] Schwobel B Jordan S Vanisaveth V Phetsouvanh R Christophel EM Phompida S Von Sonnenburg F Jelinek T Therapeutic efficacy of chloroquine plus sulphadoxine/pyrimethamine compared with monotherapy with either chloroquine or sulphadoxine/pyrimethamine in uncomplicated *Plasmodium falciparum *malaria in Laos Trop Med Int Health 2003 8 19 24 10.1046/j.1365-3156.2003.00977.x 12535245

[B11] Ndyomugyenyi R Magnussen P Clarke S The efficacy of chloroquine, sulfadoxine-pyrimethamine and a combination of both for the treatment of uncomplicated *Plasmodium falciparum *malaria in an area of low transmission in western Uganda Trop Med Int Health 2004 9 47 52 10.1046/j.1365-3156.2003.01167.x 14728606

[B12] Baird JK Basri H Jones TR Purnomo Bangs MJ Ritonga A Resistance to antimalarials by *Plasmodium falciparum *in Arso PIR, Irian Jaya, Indonesia Am J Trop Med Hyg 1991 44 640 4 1858967 10.4269/ajtmh.1991.44.640

[B13] Murphy GS Basri H Purnomo Andersen EM Bangs MJ Mount DL Gorden J Lal AA Purwokusumo AR Harjosuwarno S Sorensen K Hoffman SL Vivax malaria resistant to treatment and prophylaxis with chloroquine Lancet 1993 341 96 100 10.1016/0140-6736(93)92568-E 8093414

[B14] Taylor WR Widjaja H Richie TL Basri H Ohrt C Tjitra Taufik E Jones TR Kain KC Hoffman SL Chloroquine/doxycycline combination versus chloroquine alone, and doxycycline alone for the treatment of *Plasmodium falciparum *and *Plasmodium vivax *malaria in northeastern Irian Jaya, Indonesia Am J Trop Med Hyg 2001 64 223 8 11463107 10.4269/ajtmh.2001.64.223

[B15] Maguire JD Krisin Marwoto H Richie TL Fryauff DJ Baird JK Mefloquine is highly efficacious against chloroquine-resistant *Plasmodium vivax *malaria and *P. falciparum *malaria in Papua, Indonesia Clin Inf Dis 2006 42 1067 72 10.1086/501357 16575721

[B16] Djimde A Doumbo OK Cortese JF Kayentao K Doumbo S Diourte Y Dicko A Su XZ Nomura T Fidock DA Wellems TE Plowe CV Coulibaly D A molecular marker for chloroquine-resistant falciparum malaria NEJM 2001 344 257 263 10.1056/NEJM200101253440403 11172152

[B17] Walter RD Konigk E [*Plasmodium chaubadi *: enzymatic synthesis of dihydropteroate and its inhibition by sulfonamides](in German) Z Tropenmed Parasitol 1971 22 256 259 5134866

[B18] Ferone R Burchall JJ Hitchings GH *Plasmodium berghei *dihydrofolate reductase. Isolation, properties, and inhibition by antifolates Mol Pharmacol 1969 5 49 59 4392112

[B19] Cowman AF Morry MJ Biggs BA Cross GA Foote SJ Amino acid changes linked to pyrimethamine resistance in the dihydrofolate reductase-thymidylate synthase gene of *Plasmodium falciparum *Proc Natl Acad Sci USA 1988 85 9109 9113 10.1073/pnas.85.23.9109 3057499 PMC282673

[B20] Peterson DS Walliker D Wellems TE Evidence that a point mutation in dihydrofolate reductase-thymidylate synthase confers resistance to pyrimethamine in falciparum malaria Proc Natl Acad Sci USA 1988 85 9114 9118 10.1073/pnas.85.23.9114 2904149 PMC282674

[B21] Triglia T Wang P Sims PF Hyde JE Cowman AF Allelic exchange at the endogenous genomic locus in *Plasmodium falciparum *proves the role of dihydropteroate synthase in sulfadoxine-resistant malaria EMBO J 1998 17 3807 3815 10.1093/emboj/17.14.3807 9669998 PMC1170716

[B22] Auliff A Wilson DW Russell B Gao Q Chen N Anh le N Maguire J Bell D O'Neil MT Cheng Q Amino acid mutations in *Plasmodium vivax *DHFR and DHPS from several geographical regions and susceptibility to antifolate drugs Am J Trop Med Hyg 2006 75 617 621 17038682

[B23] White NJ Preventing antimalarial drug resistance through combinations Drug Resist Updat 1998 1 3 9 10.1016/S1368-7646(98)80208-2 17092790

[B24] Bell D Bryan J Cameron A Foley D Pholsyna K Salinity tolerance of Anopheles farauti Laveran sensu stricto P N G Med J 1999 42 5 9 11061001

[B25] Foley DH Meek SR Bryan JH The *Anopheles punctulatus *group of mosquitoes in the Solomon Islands and Vanuatu surveyed by allozyme electrophoresis Med Vet Entomol 1994 8 340 350 10.1111/j.1365-2915.1994.tb00098.x 7841489

[B26] World Health Organization Assessment of therapeutic efficacy of antimalarial drugs for uncomplicated falciparum malaria. Version 3, Draft March 3, 2001 2001 Geneva, Division of Tropical Diseases Control, WHO

[B27] Baird JK Wiady I Fryauff DJ Sutanihardja MA Leksana B Widjaya H Kysdarmanto Subianto B In vivo resistance to chloroquine by *Plasmodium vivax *and *Plasmodium falciparum *at Nabire, Irian Jaya, Indonesia Am J Trop Med Hyg 1997 56 627 31 9230793 10.4269/ajtmh.1997.56.627

[B28] Cheng Q Saul A Sequence analysis of the apical membrane antigen I (AMA-1) of *Plasmodium vivax *Mol Biochem Parasitol 1994 65 183 187 10.1016/0166-6851(94)90127-9 7935625

[B29] Felger I Tavul L Beck HP *Plasmodium falciparum *: a rapid technique for genotyping the merozoite surface protein 2 Exp Parasitol 1993 77 372 375 10.1006/expr.1993.1094 7901048

[B30] Tjitra E Baker J Suprianto S Cheng Q Anstey NM Therapeutic efficacies of artesunate-sulfadoxine-pyrimethamine and chloroquine-sulfadoxine-pyrimethamine in vivax malaria pilot studies: relationship to *Plasmodium vivax *dhfr mutations Antimicrob Agents Chemother 2002 46 3947 3953 10.1128/AAC.46.12.3947-3953.2002 12435700 PMC132782

[B31] Korsinczky M Fischer K Chen N Baker J Rieckmann K Cheng Q Sulfadoxine resistance in *Plasmodium vivax *is associated with a specific amino acid in dihydropteroate synthase at the putative sulfadoxine-binding site Antimicrob Agents Chemother 2004 48 2214 2222 10.1128/AAC.48.6.2214-2222.2004 15155224 PMC415609

[B32] Rogers WO Atuguba F Oduro AR Hodgson A Koram KA Clinical case definitions and malaria vaccine efficacy J Infect Dis 2006 193 467 473 10.1086/499314 16388497

[B33] Baird JK Wiady I Fryauff DJ Sutanihardja MA Leksana B Widjaya H Kysdarmanto Subianto B In vivo resistance to chloroquine by *Plasmodium vivax *and *Plasmodium falciparum *at Nabire, Irian Jaya, Indonesia Am J Trop Med Hyg 1997 56 627 631 9230793 10.4269/ajtmh.1997.56.627

[B34] Bojang KA Schneider G Forck S Obaro SK Jaffar S Pinder M Rowley J Greenwood BM A trial of Fansidar plus chloroquine or Fansidar alone for the treatment of uncomplicated malaria in Gambian children Trans R Soc Trop Med Hyg 1998 92 73 76 10.1016/S0035-9203(98)90962-2 9692160

[B35] Talisuna AO Nalunkuma-Kazibwe A Bakyaita N Langi P Mutabingwa TK Watkins WW Van Marck E D'Alessandro U Egwang TG Efficacy of sulphadoxine-pyrimethamine alone or combined with amodiaquine or chloroquine for the treatment of uncomplicated falciparum malaria in Ugandan children Trop Med Int Health 2004 9 222 229 10.1046/j.1365-3156.2003.01187.x 15040559

[B36] Sowunmi A Fehintola FA Adedeji AA Falade AG Falade CO Akinyinka OO Oduola AM Comparative efficacy of chloroquine plus chlorpheniramine alone and in a sequential combination with sulfadoxine-pyrimethamine, for the treatment of acute, uncomplicated, falciparum malaria in children Ann Trop Med Parasitol 2000 94 209 217 10.1080/00034980050006375 10884864

[B37] Sutanto I Supriyanto S Ruckert P Purnomo Maguire JD Bangs MJ Comparative efficacy of chloroquine and sulfadoxine-pyrimethamine for uncomplicated *Plasmodium falciparum *malaria and impact on gametocyte carriage rates in the East Nusatenggara province of Indonesia Am J Trop Med Hyg 2004 70 467 473 15155977

[B38] Puta C Manyando C Enhanced gametocyte production in Fansidar-treated *Plasmodium falciparum *malaria patients: implications for malaria transmission control programmes Trop Med Int Health 1997 2 227 229 10.1046/j.1365-3156.1997.d01-267.x 9491100

[B39] Sakihama N Kaneko A Hattori T Tanabe K Limited recombination events in merozoite surface protein-1 alleles of *Plasmodium falciparum *on islands Gene 2001 279 41 48 10.1016/S0378-1119(01)00748-X 11722844

[B40] Sibley CH Hyde JE Sims PF Plowe CV Kublin JG Mberu EK Cowman AF Winstanley PA Watkins WM Nzila AM Pyrimethamine-sulfadoxine resistance in *Plasmodium falciparum *: what next? Trends Parasitol 2001 17 582 588 10.1016/S1471-4922(01)02085-2 11756042

[B41] Pinichpongse S Doberstyn EB Cullen JR Yisunsri L Thongsombun Y Thimasarn K An evaluation of five regimens for the outpatient therapy of falciparum malaria in Thailand 1980-81 Bull World Health Organ 1982 60 907 912 6761005 PMC2535972

[B42] Kaneko A Taleo G Kalkoa M Yaviong J Reeve PA Ganczakowski M Shirakawa C Palmer K Kobayakawa T Björkman A Malaria epidemiology, glucose 6-phosphate dehydrogenase deficiency and human settlement in the Vanuatu Archipelago Acta Trop 1998 70 285 302 10.1016/S0001-706X(98)00035-7 9777715

